# Integrating Interpretability in Machine Learning and Deep Neural Networks: A Novel Approach to Feature Importance and Outlier Detection in COVID-19 Symptomatology and Vaccine Efficacy

**DOI:** 10.3390/v16121864

**Published:** 2024-11-29

**Authors:** Shadi Jacob Khoury, Yazeed Zoabi, Mickey Scheinowitz, Noam Shomron

**Affiliations:** 1 Faculty of Medical and Health Sciences, Tel Aviv University, Tel Aviv 6997801, Israel; shadekhoury@gmail.com (S.J.K.);; 2 Department of Biomedical Engineering, Faculty of Engineering, Tel Aviv University, Tel Aviv 6997801, Israel; 3 Edmond J Safra Center for Bioinformatics, Tel Aviv University, Tel Aviv 6997801, Israel; 4 Tel Aviv University Innovation Laboratories (TILabs), Tel Aviv 6997801, Israel

**Keywords:** COVID-19, vaccine efficacy, machine learning, interpretability, feature importance

## Abstract

In this study, we introduce a novel approach that integrates interpretability techniques from both traditional machine learning (ML) and deep neural networks (DNN) to quantify feature importance using global and local interpretation methods. Our method bridges the gap between interpretable ML models and powerful deep learning (DL) architectures, providing comprehensive insights into the key drivers behind model predictions, especially in detecting outliers within medical data. We applied this method to analyze COVID-19 pandemic data from 2020, yielding intriguing insights. We used a dataset consisting of individuals who were tested for COVID-19 during the early stages of the pandemic in 2020. The dataset included self-reported symptoms and test results from a wide demographic, and our goal was to identify the most important symptoms that could help predict COVID-19 infection accurately. By applying interpretability techniques to both machine learning and deep learning models, we aimed to improve understanding of symptomatology and enhance early detection of COVID-19 cases. Notably, even though less than 1% of our cohort reported having a sore throat, this symptom emerged as a significant indicator of active COVID-19 infection, appearing 7 out of 9 times in the top four most important features across all methodologies. This suggests its potential as an early symptom marker. Studies have shown that individuals reporting sore throat may have a compromised immune system, where antibody generation is not functioning correctly. This aligns with our data, which indicates that 5% of patients with sore throats required hospitalization. Our analysis also revealed a concerning trend of diminished immune response post-COVID infection, increasing the likelihood of severe cases requiring hospitalization. This finding underscores the importance of monitoring patients post-recovery for potential complications and tailoring medical interventions accordingly. Our study also raises critical questions about the efficacy of COVID-19 vaccines in individuals presenting with sore throat as a symptom. The results suggest that booster shots might be necessary for this population to ensure adequate immunity, given the observed immune response patterns. The proposed method not only enhances our understanding of COVID-19 symptomatology but also demonstrates its broader utility in medical outlier detection. This research contributes valuable insights to ongoing efforts in creating interpretable models for COVID-19 management and vaccine optimization strategies. By leveraging feature importance and interpretability, these models empower physicians, healthcare workers, and researchers to understand complex relationships within medical data, facilitating more informed decision-making for patient care and public health initiatives.

## 1. Introduction

The COVID-19 pandemic has posed unprecedented challenges to healthcare systems worldwide, necessitating the rapid development of treatments and vaccines. SARS-CoV-2, the virus responsible for COVID-19, manifests a broad range of symptoms, including fever, cough, shortness of breath, sore throat, and loss of smell, which are among the most commonly observed symptoms. Based on these symptoms, patients are categorized into different disease severity groups [1]. After symptom relief, patients are generally considered protected from reinfection for several months due to the immunological memory of the humoral and cellular immune systems [2].

Understanding the role these symptoms play in identifying and managing patients with SARS-CoV-2 infection is critical. Researchers have established a connection between certain symptoms and the severity of COVID-19 infections [3,4,5]. Moreover, these symptoms provide valuable insights into the complex interactions between the virus and the immune system. Notably, there is a significant overlap between COVID-19 symptoms and systemic symptoms that occur shortly after vaccination [6,7], which can serve as indicators of vaccine efficacy. However, immunity to SARS-CoV-2 typically does not develop immediately after vaccination, with effective protection generally beginning around day 12 [7]. Quarantining and testing every individual experiencing systemic symptoms post-vaccination would be difficult, costly, and labor-intensive, yet such measures might be necessary if SARS-CoV-2 infection cannot be robustly excluded.

The Israeli Ministry of Health publicly released data on individuals tested for SARS-CoV-2 via RT-PCR assays of nasopharyngeal swabs [8]. During the initial months of the COVID-19 pandemic in Israel, diagnostic tests adhered to Ministry of Health criteria, which were periodically updated. These criteria included factors such as symptom severity, exposure to confirmed COVID-19 cases, specific geographic locations, and the risk of complications upon infection. All tested individuals had clear indications for testing, minimizing referral bias within the dataset. Both positive and negative cases were confirmed using RT-PCR assays. Building on other works like Zoabi et al. (2020) [9], which demonstrated the dataset’s predictive power, our study further refines these models and enhances feature interpretation. Similar symptom-based predictive methods [10] have validated this approach, reinforcing the effectiveness of our methodology.

Additionally, the Ministry of Health released data on post-vaccination symptoms, revealing that many individuals exhibited side effects after receiving the vaccine. Some of these side effects were severe enough to require further medical attention or even hospitalization [11].

Interpretable machine learning models can play a crucial role in analyzing symptom patterns, immune responses, and clinical outcomes. The ability to explain the predictions made by these models in understandable terms—referred to as ML interpretability—is increasingly important in healthcare applications. For ML models dealing with clinical data and decision-making, interpretability involves presenting predictions in a way that reveals the relationship between input features and predicted outcomes. Reliable and easily understood explanations are key to gaining human trust and enabling effective ML usage [12,13,14,15]. In critical health situations, institutions tend to prefer explainable models over complex “black box” models, even if the latter are slightly more accurate [16]. In medical applications like analyzing COVID-19 data, interpretability is as crucial as traditional performance metrics like accuracy. Interpretable models are particularly valuable for predicting COVID-19 outcomes, treatment paths, and vaccine responses, where understanding the reasoning behind predictions is essential. However, with a growing array of interpretability techniques available, selecting the optimal approach for a specific use case remains a challenge.

Researchers have explored various approaches to predict COVID-19 cases based on symptoms, employing a range of methods and classifiers, including ensemble methods, gradient boosting, clustering, KNN, and deep learning techniques like LSTM and neural networks [5,17,18,19,20,21]. Some even developed their own algorithms [22]. These studies aimed to compare different prediction methods to determine which classifiers were best suited for the task. While they successfully made predictions, few studies examined the factors influencing these models and whether the predictions were sensible from all perspectives. This lack of analysis stems from the challenge of selecting appropriate interpretation methods to explain the classifiers and what those interpretations reflect.

In this paper, we address the challenge of selecting suitable interpretation techniques for machine learning models in healthcare, particularly in the context of analyzing COVID-19 data. Building on the importance of ML interpretability, we propose using machine learning models, specifically a gradient boosting model and a neural network (NN), to predict positive SARS-CoV-2 infection in RT-PCR tests based on eight fundamental questions. We employ state-of-the-art feature importance methods from both global and local techniques to comprehensively understand the factors influencing our models and their predictions, enabling users to discern the vital features in this prediction model. This approach offers new insights, aiding in the interpretation and application of the results.

Furthermore, our research provides healthcare providers with a valuable tool to enhance diagnostic accuracy and decision-making in COVID-19 testing. By training the model and applying combined global and local interpretability methods to a basic dataset from diverse populations in Israel, we ensure that our findings are applicable across different demographics. Since the symptoms analyzed, such as cough, sore throat, fever, and headache, are common worldwide, our model’s predictions remain relevant regardless of geographical location or demographic characteristics.

## 2. Methods

We employed a comprehensive approach involving multiple processes of data filtration and sorting through several pipelines. We began by carefully analyzing the dataset characteristics before developing two predictive classifiers: one using gradient boosting decision trees (GBDT) techniques and the other employing deep learning neural network architecture, both incorporating state-of-the-art methodologies. After constructing and validating these classifiers, we established a pipeline to compare advanced feature importance techniques from both global and local interpretation methods. These techniques included SHapley Additive exPlanations (SHAP) [10,23,24], LIME [12], Diverse Counterfactual Explanations (DiCE) [25,26], LightGBM (LGBM) [27,28], gradients [29], activation maximization [30], and sensitivity permutation [31], among others. This pipeline was crucial in identifying the top K important features across all nine methods, providing a holistic understanding of our model development and evaluation process. Integrating these advanced techniques allowed us to gain deep insights into the models’ inner workings and the key factors driving their predictive power.

We also applied advanced optimization techniques such as hyperparameter tuning, SMOTE (Synthetic Minority Over-sampling Technique) [32], standard scaling, logistic regression, and cross-validation to ensure optimal model performance. This meticulous approach not only improved the accuracy and robustness of our models but also provided a framework for reproducibility and scalability in similar predictive modeling tasks.

To evaluate the performance of our models, we considered key metrics, including AUC (Area Under the Curve), accuracy, sensitivity, specificity, and F1-score. These metrics offered a comprehensive assessment of the models’ performance across various classification aspects. Additionally, we employed bootstrapping methodologies to generate confidence intervals with a 5% margin of error, ensuring the reliability of our results. This evaluation process enabled us to assess the models’ performance with statistical confidence and identify areas for further improvement.

### 2.1. Data Processing and Modelling

In our data processing workflow, we adopt two distinct paths, each tailored for specific modeling approaches: Method A for Gradient Boosted Decision Trees and Method B for Deep Learning Neural Networks. Method A follows standard steps such as data loading, cleaning, converting columns to float types, and handling missing values by removing rows with NaNs. Class imbalances are addressed using SMOTE oversampling [28], followed by constructing a pipeline that leverages logistic regression for model building and evaluation.

**Figure 1:**
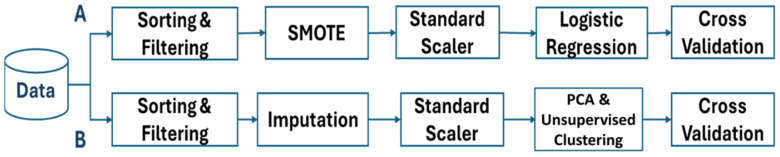
Schematic workflow of data processing. The Figure illustrates two distinct branches (A and B) in the data processing workflow. Both branches begin with sorting and filtering the data to ensure it meets the required standards for further analysis.

Method B introduces a novel CNN-based imputer, the ImputerNN class [33], which we developed to handle missing data. This imputer learns to impute missing values by training a neural network on data transformed into PyTorch tensors, with NaNs replaced by zeros. The imputation process is unique in that it treats missing data as a learning task, enabling the model to better capture underlying patterns. In comparative tests, the CNN-based imputation outperformed traditional methods like mean, median, and k-NN imputation, providing more accurate and consistent results. After imputation, the data are standardized, reduced in dimensionality using PCA, and clustered using a combination of fuzzy c-means and K-Means [25,34,35], revealing complex patterns. The novelty in this approach lies in the specific integration of neural network-based imputation with clustering techniques, optimizing the data for both Gradient Boosted Decision Trees (GBDT) and neural network models, as shown in Figure 1.

### 2.2. Study Setting and Data

The Israeli Ministry of Health has publicly released data on individuals tested for SARS-CoV-2 using RT-PCR assays of nasopharyngeal swabs. This dataset includes daily records of all residents tested for COVID-19 across the country. In addition to test dates and results, the dataset provides various details such as clinical symptoms, gender, and a binary indicator for individuals aged 60 years or older. Using this dataset, we developed a model to predict COVID-19 test outcomes based on eight binary features: gender, age 60 years or older, known contact with a confirmed case, and five initial clinical symptoms [8]. Our training set consisted of 51,831 tested individuals (4769 of whom were confirmed to have COVID-19) from the period 22 March 2020 through 31 March 2020. The test set contained data from the subsequent weeks, April 1 through April 7 (47,401 tested individuals, 3624 of whom were confirmed to have COVID-19).

### 2.3. Characteristics of the Dataset and Features

**Figure 2:**
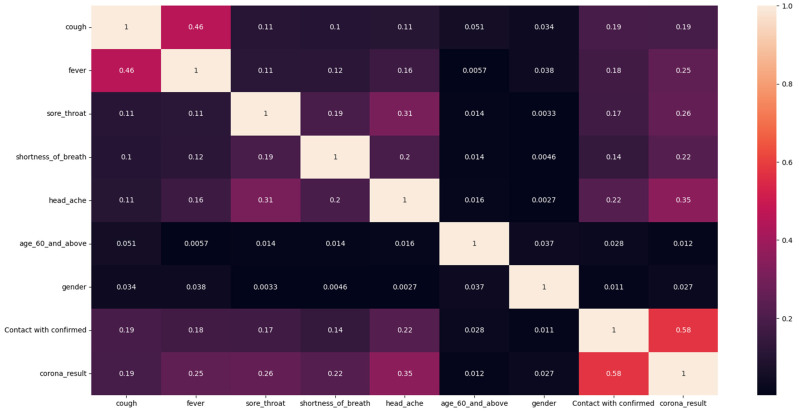
Correlation analysis plot of COVID-19 dataset. The first five features represent reported symptoms, while the other features include demographics like age and gender. “Contact with confirmed” indicates whether an individual had contact with someone confirmed to have COVID-19. Correlation values near +1 indicate strong positive relationships, while values near −1 indicate strong negative relationships.

There is no significant correlation (defined as correlation values exceeding 70%) between the features (Figure 2). This lack of correlation indicates that the features do not significantly influence each other, which mitigates potential issues with feature importance methods. In previous work, we noted that even in models with low performance, correlation served as a key indicator for certain methods, particularly those from global domains like SHAP, where correlations between features can introduce biases [36]. Ensuring the absence of bias in feature importance scores is essential when comparing interpretability methods, as it enables a fair evaluation of each method’s feature importance scores and their contribution to the model’s interpretability, leading to more reliable and accurate insights. To further understand the relation between the features and the forecast, a Probability Density Function (PDF) plot was generated.

**Figure 3:**
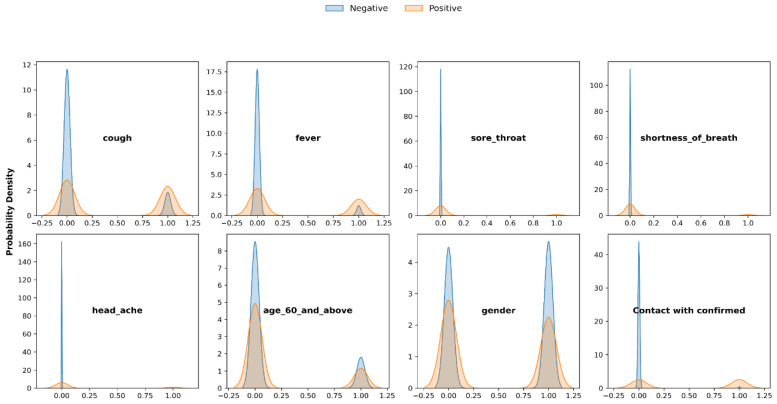
Probability Density Function (PDF) analysis of COVID-19 features in relation to the label. The plots compare the probability density of different COVID-19 features, such as symptoms and demographic factors, in relation to PCR test results (negative or positive). The blue curves represent individuals with negative PCR test results, while the orange curves represent those with positive results. Each subplot highlights the distribution of a specific feature (e.g., cough, fever, age) between the two groups, showing differences in symptom presence and other characteristics among those who tested positive or negative for COVID-19.

When looking into the PDF analysis in relation to the label, the *y*-axis represents the density probability of each feature, while the *x*-axis denotes the COVID PCR result, where a value of 1 indicates a positive PCR result (presence of COVID infection), and 0 indicates the absence of infection. It is evident that most features are associated with a low probability of identifying a positive label, suggesting these features may act as protective factors or negative predictors for COVID-19. These factors significantly reduce the likelihood of an individual having the infection. Exceptions include gender, where a value of 1 indicates female, and age above 60, which shows a high probability of both positive and negative COVID results. Three features, sore throat, shortness of breath, and headache, exhibited very low probability densities when their value was 1 and the label was positive. This indicates that it is rare for these features to have a value of 1 when the label is positive, leading us to consider these features as potential outliers. Outliers are data points that significantly deviate from the rest of the observations. In this case, these features deviate from the typical pattern observed in the dataset, making them notable as unusual instances. To further examine the relationship between each feature and the label, we analyzed the count and percentages of values associated with the label for each feature (Figure 3).

To facilitate a clearer understanding of these relationships, we present a table summarizing the counts associated with the COVID-19 label for a feature “Sore Throat”. This tabular representation will highlight the distribution of symptoms in relation to COVID-19 outcomes, making it easier to discern patterns and correlations within the data.

From the analysis (Table 1), it is notable that individuals with a sore throat are more likely to test positive for COVID-19 compared to those without a sore throat. The percentage of positive cases among those with a sore throat (about 91%) is significantly higher than those without a sore throat (about 7%).

In order to examine the relationship between each feature and the label, we analyzed the count and percentages of values associated with the label for each feature. It is evident that all features show a higher count of 0 compared to 1, where 1 indicates the presence of the symptom or condition (Figure S1). This suggests that most individuals in the study do not exhibit these specific symptoms or conditions. The higher count of 0 indicates a lower prevalence of the symptoms or conditions associated with these features within the studied population. This information helps us understand the distribution and prevalence of these characteristics within the context of the study.

To investigate this further, we examined the percentages of 0 and 1 values, including those for the label.

From the analysis (Table 2), it is notable that the majority of values show a low percentage of 1, indicating a greater prevalence of absence rather than the presence of the specific feature. Even more striking is that instances labeled as positive account for only 5% of the total. Additionally, symptoms such as sore throat, shortness of breath, and headache were observed in less than 1% of cases, suggesting these may be anomalies or rare occurrences within the dataset. These findings highlight the need for further investigation into these symptoms as potential indicators of the condition being studied.

To better understand the behavior of these symptoms, we analyzed the symptomatology of each patient by examining the count of symptoms exhibited. We also assessed the frequency of each symptom being present, denoted by a value of 1, across the entire patient cohort.

**Figure 4:**
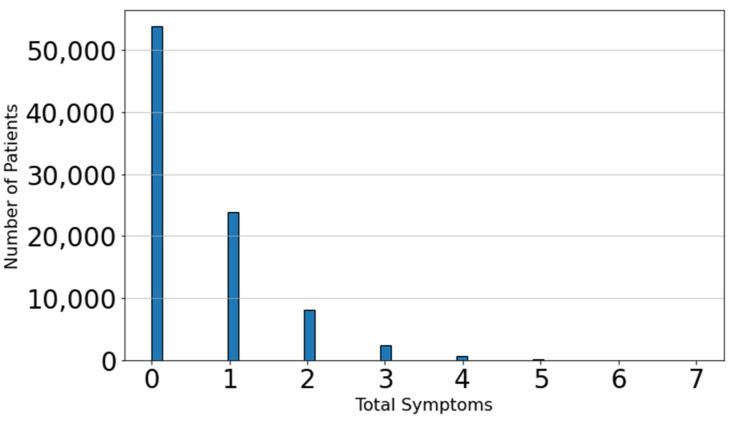
Histogram of total active symptoms per patient. The *x*-axis represents the number of symptoms, while the *y*-axis shows the frequency of patients exhibiting that number of symptoms.

The fact that most patients did not exhibit any symptoms (Figure 4) suggests that these individuals either did not have COVID-19 or were in the early stages of the disease. Conversely, a smaller subset of patients presented with at least one symptom, with the highest number of symptoms reported by a single patient being seven. To gain deeper insights into the subset of patients with active symptoms, we focused on these patients and analyzed which symptoms were most frequently present.

**Figure 5:**
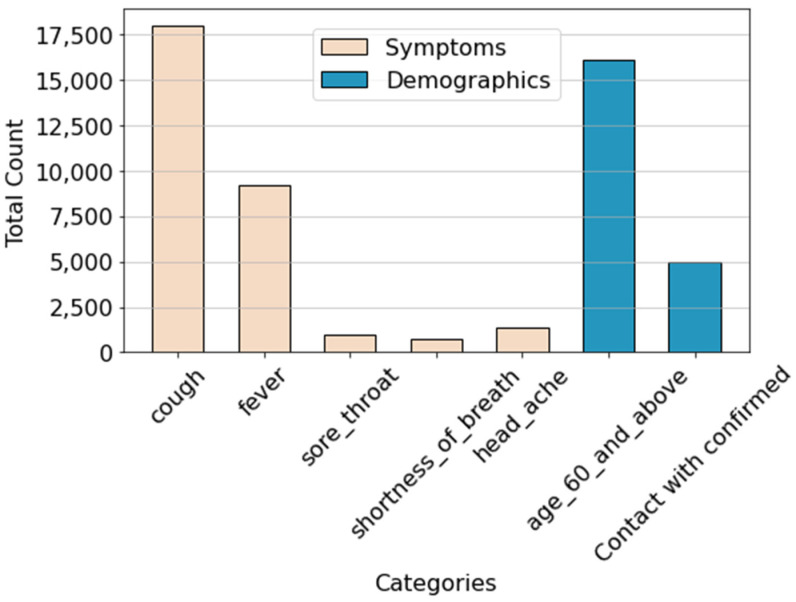
Total count of symptoms experienced by patients. The *x*-axis represents different symptoms as labeled. The orange bars indicate the count of symptoms experienced by patients, while the blue bars represent demographic data related to the symptoms.

Patients with at least one active symptom predominantly reported experiencing cough, being over 60 years old, and having a fever (Figure 5). This observation highlights significant trends among symptomatic patients, indicating that these factors are prevalent in individuals exhibiting symptoms of COVID-19.

Additionally, among the seven symptoms analyzed, three—sore throat, shortness of breath, and headache—stood out. The occurrence of these symptoms is significantly lower compared to the other four symptoms, each with fewer than 2500 reported instances. This disparity underscores a notable gap in the frequency of these symptoms, suggesting a lower incidence of sore throat, shortness of breath, and headache relative to the other symptoms surveyed. Moreover, this lower incidence might indicate that patients exhibiting these symptoms belong to a distinct subgroup. These patients could be considered potential outliers in the context of symptom distribution, as their clinical presentations deviate from the more commonly observed symptoms., which warrants further investigation into their reporting accuracy or possible association with unique patient characteristics. Further exploration of the factors contributing to this discrepancy could provide valuable insights into their association with the underlying condition or the reporting tendencies of individuals.

### 2.4. Development of the Model

In our model development, we employed two distinct yet complementary approaches: a Gradient Boosting Decision Trees (GBDT) built with LGBM [37,38] and a deep learning neural network featuring a bottleneck layer (Figure S2). The gradient-boosting model, a strong predictor in tabular data prediction, handles missing values seamlessly while providing valuable insights into feature importance. On the other hand, our neural network, with its multiple hidden layers with diverse activation functions, notably tanh and relu, and the deliberate inclusion of a bottleneck layer [39], offered a deeper understanding of complex patterns within the data. This architectural choice not only regulated information flow and controlled model complexity but also refined interpretability by focusing on the most impactful features.

By combining these approaches, we harnessed the predictive power of gradient boosting and the deeper connections of deep learning, creating a model that balances simplicity, interpretability, and the ability to capture intricate data relationships. This hybrid model allowed us to capitalize on the strengths of each approach: the gradient-boosting machine for its robust predictive performance and the neural network for its ability to uncover hidden patterns and feature importance. The result is a comprehensive predictive model that not only delivers accurate predictions but also provides a deeper understanding of the underlying factors driving those predictions, offering valuable insights for decision-making in our domain.

To identify the principal features driving the model prediction, we turned to various methods of feature importance analysis. Among these, feature attributions and counterfactual explanations (relating to or expressing what has not happened or is not the case) are popular approaches to explaining ML models. The former assigns an importance score to each input feature, while the latter provides input examples with minimal changes to alter the model’s predictions. To understand the difference between the algorithms, we normalized the feature scores and analyzed the difference between them; we also set a threshold on the feature score depending on the relevance of that feature and depending on the dataset used to calculate that feature score. The normalization was performed using the following Equation (1):

(1) $$\begin{array}{c} {\bar{S}}_{i}=\frac{s_{I}}{\underset{i}{\max}S} \end{array}$$

where ${\bar{S}}_{i}$ is the normalized score of feature i, and Si is the score of feature i. We utilized this normalization technique to grasp the significance of a feature in relation to others within the dataset without diving into the mathematical algorithms behind each method.

**Figure 6:**
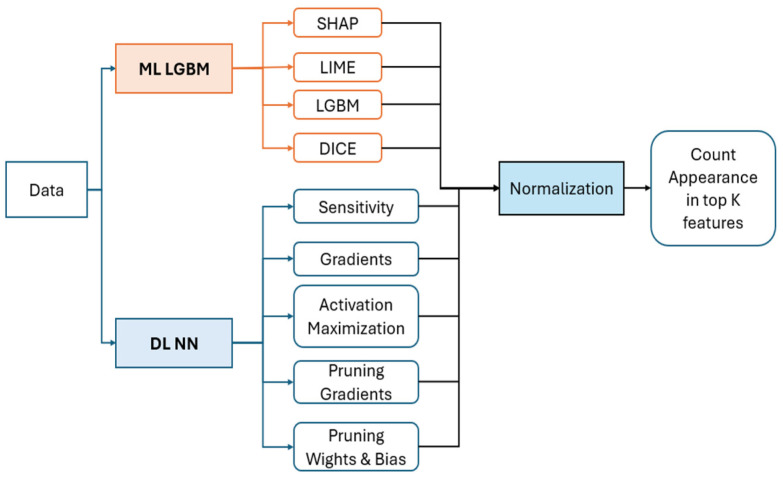
A schema of feature importance extraction. This schema depicts the process of feature importance extraction using two models: LGBM and Deep Learning Neural Network (DL NN). Each model is paired with its corresponding interpretation method to assess feature importance.

In our process, we first pass the data through prediction steps and then apply algorithms for feature importance analysis. After obtaining the feature importance scores, we normalize these scores using the provided equation [7]. Following normalization, we carefully examine each of the normalized values by counting the occurrences of each feature within the top K scores. For example, when selecting the top K features, we tally how frequently each feature appears among these top K normalized scores. Figure 6 illustrates the workflow used to extract and analyze feature importance, demonstrating the systematic steps involved in this approach.

### 2.5. Evaluation of the Model

The model’s performance on the test set was evaluated using the area under the receiver operating characteristic curve (auROC). Additionally, precision–recall curves, which depict positive predictive value (PPV) versus sensitivity, were plotted across various thresholds. Metrics including sensitivity, specificity, PPV, negative predictive value, false-positive rate, false-negative rate, false discovery rate, and overall accuracy were computed for all thresholds from the ROC curves. To estimate the uncertainty around these performance measures, confidence intervals (CI) were derived using the bootstrap percentile method with 5000 repetitions [30,40]. This resampling technique provides a robust means of assessing the model’s performance across a range of thresholds, offering valuable insights into its predictive capabilities.

### 2.6. Comparison of Interpretation Methods

#### 2.6.1. Global Interpretation Methods

##### The First Method Is Shapely Additive Explanations (ShAP)

In ShAP, we estimate how important a model is by seeing how well it performs with and without that feature for every combination of features. It is important to note that Shapley Additive Explanations calculates the importance of local features for every observation [10]. It is also important to mention that the SHAP values do not provide causality. Lloyd Shapley came up with this solution concept for a cooperative game in 1953. Shapley wants to calculate the contribution of each player in a coalition game. Assume there are N players and S is a subset of the N players. Let V(S) be the total value of the S players. When player i joins the S players, Player i’s marginal contribution is v(S∪{i}) − v(S). If we take the average of the contribution over the possible different permutations in which the coalition can be formed, we obtain the right contribution of player i: [23,24]

(2) $$\phi_{i}\left(v\right)=\sum_{S\subseteq \frac{N}{i}}\frac{\left|S\right|!\left(N-\left|S\right|-1\right)!}{N!}\left(v\left(S⋃i\right)-v\left(S\right)\right)$$

##### The Second Method Is Sensitivity-Permutations Analysis

Sensitivity analysis involves systematically testing a neural network’s adaptation to minor variations in input features, providing insights into the model’s capabilities, and discerning the influence of individual features on network predictions. By gauging the network’s response to subtle changes, sensitivity analysis unveils the model’s robustness, interpretability, and the relative impact of features on its decision-making process [31].

##### The Third Method Is LightGBM Feature Importance

LightGBM is a fast Gradient Boosting framework with a built-in feature importance method that ranks the importance of each feature depending on its effects on the prediction. LightGBM feature importance calculates the average gain of the feature when it is used in a booster tree while using LightGBM as the prediction model (booster); the gain implies the relative contribution of the corresponding feature to the model calculated by taking each feature’s contribution for each tree in the model. A higher value of this metric, when compared to another feature, implies it is more important for generating a prediction. Gain is calculated for a split by subtracting the weighted entropies of each branch from the original entropy [27]. When training a Decision Tree using these metrics, the best split is chosen by maximizing the Gain. The entropy is calculated using the equation of Shannon’s entropy [28].

(3) $$\begin{array}{c} \mathrm{Entropy}=-\sum_{i}^{c}p_{i}{log}_{2}\left(p_{i}\right)\;\;\;\; \end{array}$$

In which pi is the probability of the (i) event. After calculating the entropy of each branch, the weighted split entropy is calculated by weighing the entropy of each branch by how many elements it has using the following Equation (4):

(4) $$E_{split}=\frac{R}{N}E_{R}+\frac{L}{N}E_{L}$$

in which R and L are the number of elements in the right and left branches, respectively, and N is the number of all elements within the tree, whilst $E_{R}and\;E_{L}\;$represent the entropy of each branch. After calculating the split entropy $E_{split\;}$the gain can be calculated using the following Equation [33]:

(5) $$Gain=E_{before}-E_{split\;\;\;\;\;\;\;\;\;\;\;\;\;\;\;\;\;}$$

With $E_{before}\;$ being the entropy of the tree before splitting.

##### The Fourth Method Is DiCE

Diverse Counterfactual Explanations DiCE employs counterfactual (CF) explanations, which give critical information on how to modify the outcome (prediction) of such situations by understanding the causes of a negative outcome, but this is not enough. It is also crucial to know what to do in the future to attain a better outcome (assuming that the algorithm remains relatively static).to explain it more simply, DiCE provides a “What-if” explanation for model results. Unlike other explanations that rely on estimating the classifier’s decision boundary, counterfactual (CF) explanations have the advantage of always being true in relation to the underlying model since they provide direct outputs of the algorithm. Furthermore, counterfactual examples may be interpretable by humans by allowing users to explore “What-if” possibilities like what they do in their everyday lives. Counterfactual explanations are proposed as a method for giving option perturbations that would have changed the forecast of a model. In other words, given a feature *x* as an input, the prediction yielded by an ML model f a counterfactual explanation of the contribution of the input to produce an alternate result by using a similar calculation [25]. This can be achieved using the following Equation (6):

(6) $$c={argmin}_{c}[y_{loss}(f(c),y)+|x-c|$$

where the first part ($y_{loss}$) pushes the counterfactual towards a prediction different from the original instance, and the second part keeps the counterfactual close to the original instance [26].

#### 2.6.2. Local Interpretation Methods

##### The First Method Is LIME

The goal of Local Interpretable Model-Agnostic Explanations **(**LIME) is to identify an interpretable model over the interpretable representation that is locally faithful to the set classifier. It takes any machine learning models as input and generates explanations about feature contributions in making a prediction. It assumes that it is a black box model, which means that it does not know the inner workings of models and generates an explanation based on this assumption. It is also important to note that the LIME values do not provide causality. The explanation produced by LIME is obtained by the following Equation:

(7) $$\xi \left(x\right)=\underset{\mathrm{g\epsilon G}}{\mathrm{argmin}}L\left(f,g,\pi_{x}\right)+Ω(g)$$

where $L\left(f,g,\pi_{x}\right)$ is a measure of how unfaithful g is in approximating f in the locality defined by $\pi_{x}$. Ω(g) be a measure of complexity, where the goal is to minimize the locality-aware loss $L\left(f,g,\pi_{x}\right)$ without making any assumptions about f since the explainer is supposed to be model-agnostic [12].

##### The Second Method Is Gradients—Backward Propagation

Backward propagation, or backpropagation, is a fundamental process in training neural networks. It involves iteratively adjusting the model’s weights by computing the gradient of the loss function with respect to each weight. This gradient information is then used to update the weights in the opposite direction of the gradient, minimizing the difference between the model’s predictions and the actual targets. Importantly, if the gradients are extracted during this process, they provide valuable insights into how each feature contributes to the overall output. This analysis aids in understanding the influence of individual features on the network’s predictions, enhancing our comprehension of the model’s decision-making process [29].

##### The Third Method Is the Activation Maximization Methodology

Activation maximization, often implemented through techniques like Class Activation Mapping (CAM), is a method used to visualize and understand the decision-making process of neural networks, particularly convolutional neural networks (CNNs). By maximizing the activation of specific neurons or channels in the network’s layers, CAM highlights regions in the input data that strongly contribute to the network’s prediction for a given class. This visualization technique not only aids in interpreting and localizing the features in the input that are most influential in driving the model’s decision but also sheds light on the importance of these features in the context of deep neural networks. Activation maximization, when applied to DDNs, provides valuable insights into feature importance, offering a nuanced understanding of the specific elements that play a crucial role in the network’s decision-making process during classification tasks [30].

##### The Fourth Method Is Activation Maximization Methodology with Pruning

Integrating Class Activation Mapping (CAM) with strategic pruning of the most activated neurons in each layer provides dual advantages for Deep Neural Networks (DDN). CAM enhances interpretability by visualizing critical regions in the input crucial for the network’s predictions. Simultaneously, the concurrent pruning of highly activated neurons optimizes the network by preserving essential features while discarding less informative elements. This joint approach not only streamlines the DDN for efficiency but also facilitates nuanced feature importance extraction, offering a refined understanding of the influential factors in the decision-making process. The pruning process involves creating new linear layers using weights and biases from the most activated neurons. To choose the most activated neurons, we employ two important metrics to assess the performance and behavior of our neural network model. The first metric is based on the principles of backpropagation, a fundamental algorithm in neural network training. This metric examines the gradients of the loss function with respect to the model’s weights and biases, providing insights into how adjustments to these parameters affect the overall performance and accuracy of the network. In contrast, our second metric focuses on feedforward analysis, specifically targeting the weights and biases that lead to the most significant shifts in the network’s output during the forward pass. Unlike backpropagation, which relies on gradients for parameter updates, this metric offers a perspective on the network’s behavior based solely on its feedforward pass, shedding light on the influence of specific parameters on the model’s predictions. Together, these metrics offer comprehensive insights into both the training dynamics and the structural aspects of our neural network model [41].

To summarize the differences, strengths, and weaknesses of the various methods used in our analysis, we present the following table (Table 3).

## 3. Results

Our analysis of the diverse pipelines and classifiers provides crucial insights into their performance. We assessed metrics such as AUC (Area Under the Curve), AUPRC (Area Under the Precision–Recall Curve), accuracy, and sensitivity to evaluate the effectiveness of each classifier. Comparisons with baseline models were conducted to determine the degree of improvement. Following thorough verification and evaluation of these models, we investigated the feature importance derived from both global and local interpretation methods.

This comparative examination yields valuable insights into the distinct characteristics of features, which were subsequently normalized for clear presentation. This approach not only facilitates an assessment of classifier performance but also highlights the significance of features within the predictive models.

Moreover, our study revealed intriguing patterns in performance metrics across different classifiers. For example, the AUC values were notably close, with the GBDT model achieving 0.90 and the DL model achieving 0.89, suggesting similar discrimination power between the two models, though the GBDT model had a slight advantage. The AUPRC scores underscored the trade-off between precision and recall, offering additional perspectives on model performance. Sensitivity analysis further deepened our understanding of how each model responded to changes in input variables.

In addition to evaluating model performance, we conducted an in-depth examination of the top K important features identified by each classifier. These features, derived from advanced methodologies, provided valuable insights into the underlying factors driving predictions. We applied normalization techniques to ensure fair comparison and presented these results in an interpretable manner. We also compared model performance using these top K features to assess whether the interpretation method successfully identified the most important features and if these features contributed to improved model performance compared to baseline models. Notably, we found that incorporating these key features significantly enhanced model performance, with both the gradient boosting and deep learning models surpassing the baseline in terms of predictive accuracy and robustness.

### 3.1. Prediction Model Performance

**Figure 7:**
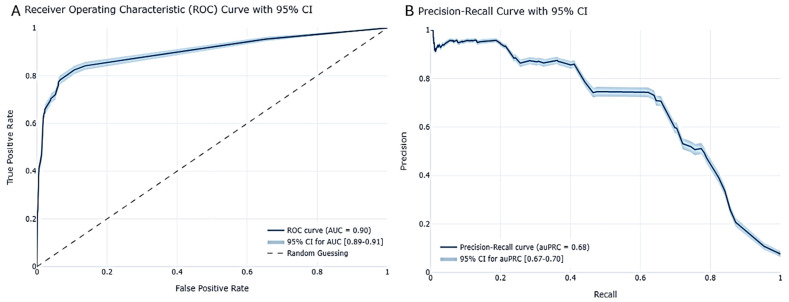
Gradient boosting LGBM model performance. (**A**) ROC curves with CI of 95% derived by bootstrapping; (**B**) Precision–Recall curve with CI of 95% derived by bootstrapping.

For the prediction test, the LGBM model demonstrated strong predictive performance, achieving an auROC (area under the receiver operating characteristic curve) of 0.90 with a 95% confidence interval (CI) ranging from 0.89 to 0.91 (Figure 7A). Additionally, the model achieved an auPRC (Area Under the Precision–Recall Curve) of 0.68, with a 95% CI spanning from 0.67 to 0.70 (Figure 7B). This indicates the model’s effective ability to discriminate between classes. Additionally, the auPRC (Area Under the Precision–Recall Curve) was calculated as 0.68, with a 95% CI ranging from 0.67 to 0.70. This highlights the model’s precision and its capacity to maintain a high positive predictive value (PPV) across varying thresholds. These results provide compelling evidence of the model’s superior performance compared to the established baseline, as shown by the improved accuracy metrics and their corresponding confidence intervals in Table 4 below. To ensure the robustness of the model and its metrics, the confidence intervals were validated using bootstrapping, further reinforcing the reliability of the model’s performance, as can be seen in the Supplementary Material (Table S1).

Our model demonstrated superior performance compared to the random baseline model across all metrics, highlighting its robust predictive power and reliability. To establish this baseline, we used multiple prediction setups without parameter tuning, allowing the model to interact with the data “out of the box” without any intervention. By averaging results from multiple runs, we generated baseline values. These meticulous steps ensured that the baseline accurately reflected the model’s performance in its initial, unoptimized state. By surpassing this baseline across all metrics, our model has proven its capability to deliver trustworthy and accurate results, instilling confidence in its predictions. This enhanced predictive performance also has significant implications for feature importance analysis, as a more accurate model inherently provides more reliable insights into feature importance.

**Figure 8:**
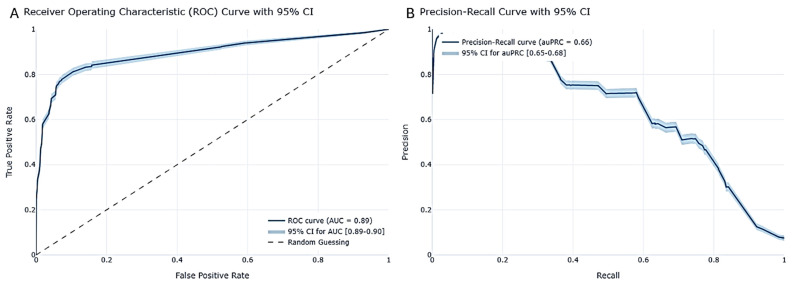
DL model performance. (**A**) ROC curves with CI of 95% derived by bootstrapping; (**B**) Precision–Recall curve with CI of 95% derived by bootstrapping.

In the prediction test, our DL model showcased robust predictive capabilities, yielding an auROC (area under the receiver operating characteristic curve) of 0.89, with a 95% confidence interval (CI) ranging from 0.89 to 0.90 (Figure 8A). This score underscores the model’s proficiency in effectively distinguishing between classes. Additionally, the model achieved an auPRC (Area Under the Precision–Recall Curve) of 0.66, with a 95% CI spanning from 0.65 to 0.68 (Figure 8B), emphasizing its precision and ability to uphold a high positive predictive value (PPV) across diverse thresholds. These results are indicative of the model’s superior performance when compared to the established baseline, as evidenced by the enhanced accuracy metrics and their respective confidence intervals, as detailed in the accompanying Table 5 below. To ensure robustness, the confidence intervals were validated using bootstrapping, further reinforcing the reliability of the model’s performance, as can be seen in the Supplementary Material (Table S2).

As observed, our DL model surpassed all performance metrics compared to the baseline model, delivering exceptional predictions with high accuracy and a strong balance between true positive and true negative labels. This outcome underscores the model’s predictive power, providing us with reliable and precise predictions that are crucial for our analysis.

The model’s performance was re-evaluated to ensure its reliability and validity, as the model relied on data reported by the Israeli Ministry of Health, which has limitations, biases, and missing information regarding some of the features. Zoabi et al. (2020) [9] established the dataset as reliable and minimized biases by ensuring that COVID-19 testing criteria were based on specific symptoms and risk factors. This helped confirm the dataset’s credibility for predictive modeling for the LGBM model. Similarly, we re-evaluated our DL model to ensure reliability and validity, addressing inherent limitations, biases, and missing data in the Israeli Ministry of Health dataset.

**Figure 9:**
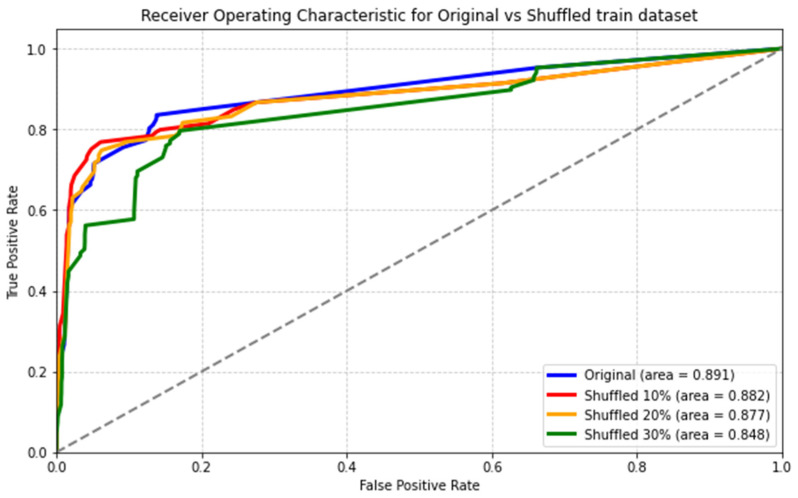
Performance of Stimulated and Shuffled Test sets. The ROC curve illustrates the performance of the model with 10%, 20%, and 30% of the dataset shuffled. The *x*-axis represents the false positive rate, and the y-axis represents the true positive rate, showing how the model’s performance varies with different levels of data shuffling.

Despite the challenges of self-reported symptoms and missing values, our model still achieved high accuracy. We designed a prospective test set by filtering out negative values for symptoms, minimizing bias in the data. When applied to this adjusted set, our DL model, with an AUC of 0.89, maintained a strong performance. This consistency under biased conditions bolsters confidence in the DL model’s robustness and suggests that the model’s performance remains stable under similar conditions (Figure 9).

Here, we can see that shuffling 10% or 20% of data had minimal impact on the model’s AUC score, with the scores remaining relatively stable at 0.891 for the original data, 0.882 for 10% shuffled data, and 0.877 for 20% shuffled data. This decrease suggests that the model’s performance becomes more sensitive to larger variations in the data distribution. Nonetheless, the overall consistency in performance across different levels of data shuffling reinforces our confidence in the model’s predictive power and its ability to generalize well.

### 3.2. Feature Importances

**Figure 10:**
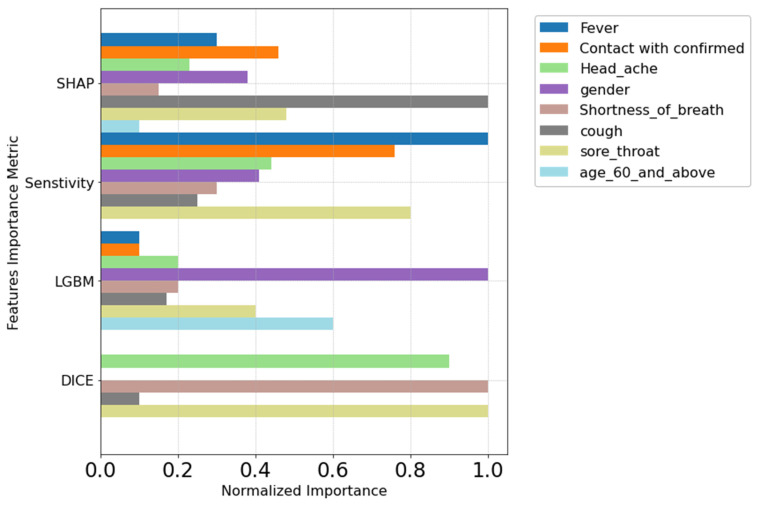
Normalized importance score of global interpretation methods. The *x*-axis represents the normalized importance scores, with 1 indicating the highest importance score for each feature. The *y*-axis lists the global interpretation methods that correspond to these scores.

After running the GBDT and DL models and inputting these models to the four different global interpretation methods, a feature importance plot was produced. As can be seen in (Figure 10) where the *y*-axis refers to the feature importance interpretation method, and the *x*-axis shows the normalized importance score as calculated by Equation (1), which shows the impact that feature has on the classifier per that method. Here, it is clear that some of the features were giving a normalized score of 1. As can be seen, cough, sore throat, and shortness of breath were predominantly with higher importance scores across multiple methods, highlighting their importance to the predictor.

**Figure 11:**
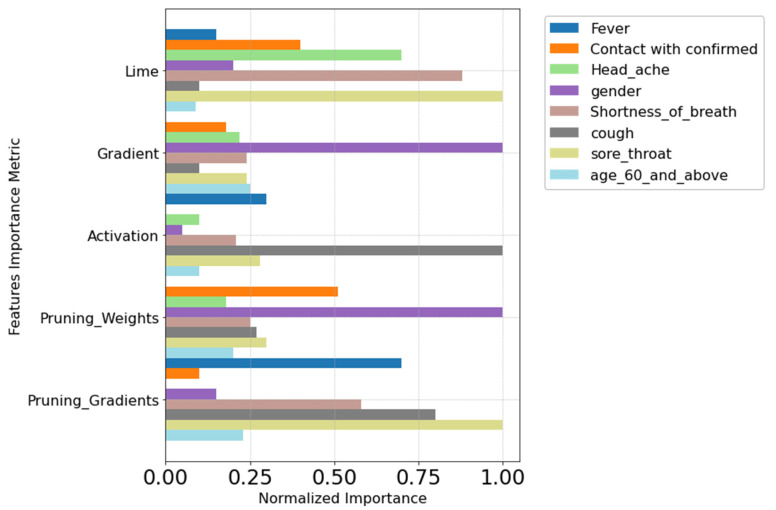
Normalized importance score of local interpretation methods. The *x*-axis represents the normalized importance scores, with 1 indicating the highest importance score for each feature. The *y*-axis lists the local interpretation methods that correspond to these scores.

Upon execution of both the DL and LGBM models and running it in the local interpretation methods, a feature importance plot was generated, as depicted in (Figure 11). The *y*-axis corresponds to the feature importance interpretation method, while the *x*-axis displays the normalized importance score computed using Equation (1). This score represents the influence each feature has on the classifier according to the respective method. Notably, several features received a normalized score of 1 in multiple methods.

Noteworthy are the features of fever, sore throat, cough, and gender, which consistently exhibited higher importance scores across various methods. This emphasizes their significance to the predictor.

### 3.3. Combined Feature Importances

Having nine different methods to assign feature importance can introduce multiple viewpoints, potentially leading to varied conclusions. To tackle this issue, we adopted a focused approach by examining the top K features, with K set to 4. By tallying how frequently each feature appeared in these top K spots across all methods, we gained valuable insights into which features held significant importance for the predictor.

**Figure 12:**
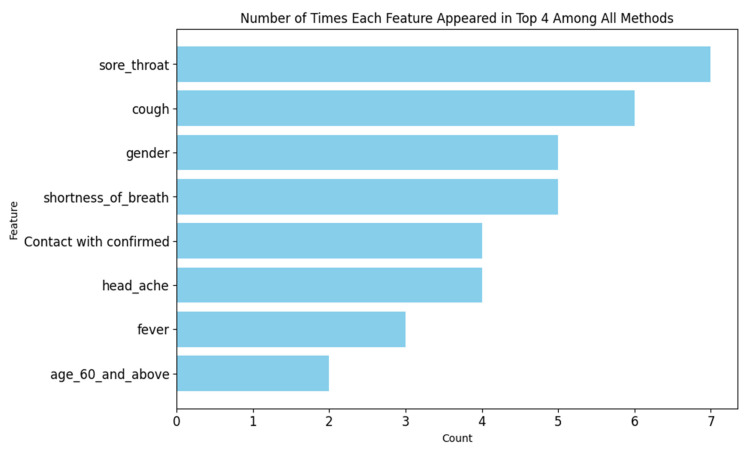
Top four features across all methods. The *y*-axis lists the features, while the *x*-axis shows the count of how many times each feature appeared in the top four spots across all interpretation methods. For instance, a count of *n* = 7 indicates that the feature appeared in the top four spots seven times across different methods.

The *y*-axis refers to the feature, and the *x*-axis refers to the count of how many times this set feature appears in the top four normalized features’ importance scores for all nine methods. As can be seen in (Figure 12), sore throat appears 7 times out of 9 in the top four features with major contributions to both the LGBM and DNN DL predictors, respectively. This count indicates that sore throat is one of the most influential features on both the predictors regardless of interpretation methodologies employed, reinforcing its pivotal role in both LGBM and DL models, followed by cough at six and gender shortness of breath with a count of 5.

To ensure that the choice of K = 4 was optimal among the range of K values from 0 to 6, we conducted a comparative analysis. This involved testing the AUC ROC score for the top 4 features from each method across various values of K. The goal was to identify the K value that provided the highest AUC for each method while maintaining the model’s performance as close to the original as possible or even surpassing the baseline constructed from multiple random runs. It is crucial to note that reducing the features to only the top K could impact the model’s ability to effectively discriminate between classes. Thus, this iterative approach allowed us to strike a balance between feature reduction and model performance enhancement. Although we observed that K = 5 and K = 6 yielded better AUC scores in specific cases, such as with SHAP and LIME, we ultimately selected K = 4 based on a comprehensive cost–benefit analysis and considerations of model complexity. This choice reflects our objective to maximize feature addition without overcomplicating the model. While higher K values may show improved AUC scores, the marginal improvements, particularly in the case of LGBM, do not justify the increased complexity and potential overfitting associated with them. Therefore, choosing K = 4 strikes an optimal balance between enhancing predictive capability and maintaining a streamlined model structure, ensuring interpretability and manageable complexity.

At K = 4, we were able to obtain an AUC score for all methods that were better than the baseline of 0.5, while methods like ShAP were able to be very close to the original AUC score of 0.90. as can be seen in (Figure S3) in the Supplementary Material.

Doing the same test on the DL method, we were able to produce an AUC plot, as can be seen in (Figure S4) in the Supplementary Material.

From this, we can see that K = 4 also in the DL keeps all AUC scores above the baseline and even some very close to the original AUC of 0.89. This result indicates that selecting the top four features consistently improves model performance, surpassing or at least maintaining baseline performance across various methodologies of feature importance scores for both the LGBM and DL predictor, respectively, showcasing that the interpretation methods were able to successfully detect the most important features influencing the predictors.

### 3.4. Post-Vaccine Symptoms

Turning our focus to the second dataset [11], which contained information on post-COVID-19 vaccination symptoms and hospitalization, it, unfortunately, did not fit within the scope of our feature importance analysis due to its limited size as the dataset included data from only 5282 individuals from the year’s 2021–2023, where we regarded anyone with no vaccine dose number as having at least one vaccine shot per the documentation provided with the data [42]. However, acknowledging this limitation, we examined the statistics generated by this dataset, and several of these statistics caught our attention.

**Figure 13:**
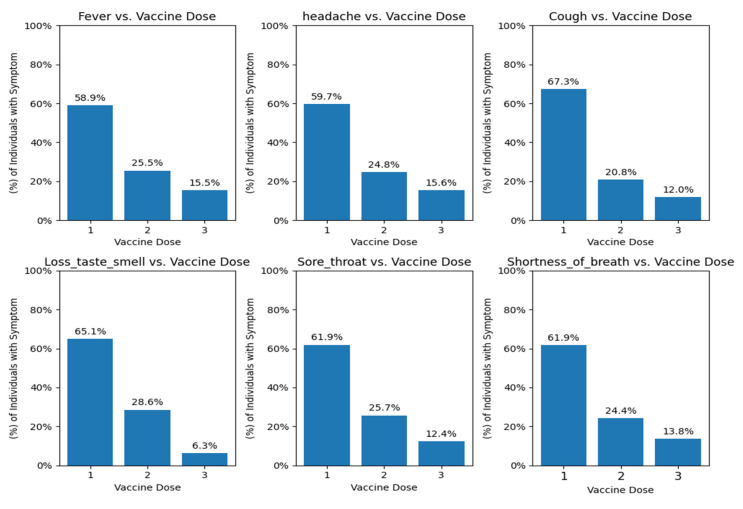
Percentage of symptom occurrence per vaccine dose. The histogram for each feature displays the percentage of reported symptoms corresponding to the number of doses taken, ranging from 1 to 3 doses. Each bar represents the percentage of symptom occurrence for each dose category.

We observe that symptoms such as fever, cough, and sore throat are present after the first vaccine dose (Figure 13). Notably, sore throat was reported as a symptom by approximately 62% of individuals following the first vaccine dose. It is also important to note that the percentage of individuals reporting sore throat decreases with each subsequent vaccine dose, similar to the trend observed for other symptoms. We further analyzed the data to determine which age groups experienced these symptoms.

We also looked at the data to analyze which age groups experienced these symptoms.

We can see that symptoms are mostly reported among young patients (under 50), where we can see sore throat being reported among the middle age group of ages 31–50 with a percentage of 26–27%, as well as shortness of breath at 25–27%, while cough is being reported in younger and older groups of ages: 21–30 and 61–70, respectively, 22% (Figure S5).

The critical analysis centered on the comparison between reported symptoms and instances of hospitalization, as this provides valuable insights into the severity and impact of post-vaccination reactions.

**Figure 14:**
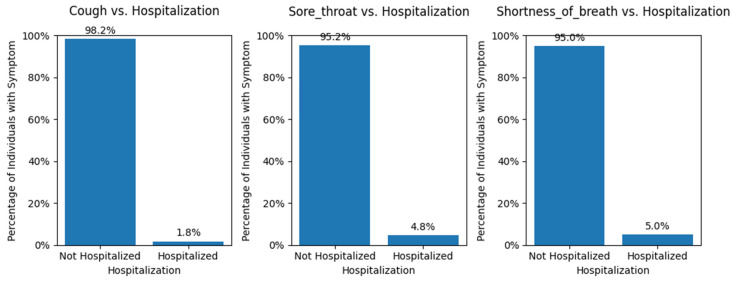
Hospitalization percentage across all individuals with reported symptoms. The *y*-axis represents the symptoms, while the *x*-axis displays the percentage of individuals with each symptom who required hospitalization.

The percentage plot where the *y*-axis is the percentage of individuals reported having that symptom, and the *x*-axis represents a split into two groups of the percent of hospitalized and non-hospitalized individuals experiencing that symptom. We can see that approximately 5% of all cases reported with sore throat or shortness of breath resulted in hospitalization (Figure 14).

## 4. Discussion

Ongoing studies are exploring the distinct pathogenesis mechanisms of SARS-CoV-2 and its spectrum of symptoms. Our approach offers a unique perspective on feature importance for COVID-19 symptoms, allowing us to identify high-importance features consistently highlighted across both global and local interpretation methods. This provided a clearer understanding of the crucial factors influencing our models and the prediction of COVID-19 using symptomology. By comparing various methodologies, we identified key features that emerged as priorities, enhancing the robustness and reliability of our model interpretation process. This approach simplified result interpretation and ensured that selected methodologies robustly pointed to influential features validated by multiple performance metrics.

Examining the AUC of both models, the DL model achieved 0.89 (Figure 11), and the LGBM model achieved 0.90 (Figure 10). The Hanley and McNeil test [43] indicated that the DL model is significantly superior, suggesting it has greater predictive power and stronger feature importance relations. Moreover, the choice of K = 4 top features was deemed adequate, addressing the challenge of the curse of dimensionality [44]. Despite initially higher AUC scores (0.90 for LGBM and 0.89 for DL), reducing features to four showed that the reduced-feature model maintained a commendable AUC exceeding 0.5 (Table 6 and Table 7). This empirical discovery challenges the anticipated loss of predictive power associated with feature reduction and dismisses concerns about overfitting beyond the initial model’s performance threshold. Hence, our research underscores the complex interplay between model simplicity and performance resilience, offering insights into the intricacies of feature importance and effect on the model predictive power and model complexity within the domain of machine learning interpretability research. Moreover, this suggests that a reduction to 4 out of 8 features strikes a balanced sufficiency, rendering the model suitable for predictive tasks, particularly in the realm of medical data analysis.

The DL model’s performance is attributed to its multi-layer structure, which enables it to uncover intricate patterns and relationships between features and predictions. In contrast, LGBM, while powerful, has limitations in capturing complex patterns. The DL deep architecture allows for more meaningful insights and better performance.

Further analysis of the models revealed that “Sore Throat” appeared five times among the top four features across all local interpretation methods and four times across global interpretation methods (Figure 12). This consistent appearance underscores “Sore Throat” as a key indicator in predictive modeling. This finding highlights the differences between feature importance methods, with Lime and Class Activation Mapping handling correlative data differently [34]. CAM’s effectiveness in recognizing complex patterns and Lime’s ability to detect outlier cases among patents validate “Sore Throat” as a significant feature, enhancing confidence in its role in predictive analysis and immune response indication.

**Figure 15:**
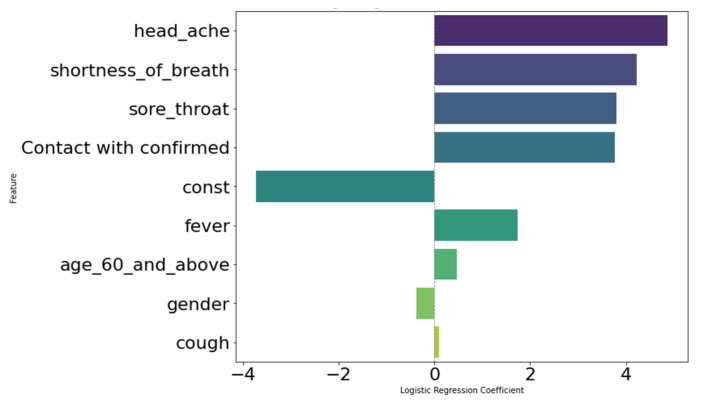
Logistic regression coefficients with label. The plot displays the coefficients of the logistic regression model, with each coefficient labeled according to its corresponding feature. The *x*-axis represents the coefficients, and the *y*-axis lists the features, illustrating the impact of each feature on the model’s predictions.

To validate these findings, we use logistic regression with 0.89 AUC and 0.95 Accuracy to determine the significance of various features in predicting the outcome. The extracted coefficients from the logistic regression model serve as our ground truth, as can be seen in the Figure below (Figure 15).

The regression coefficients used in our analysis are logistic regression coefficients, which are particularly suited for binary features that represent the change in the log odds of the dependent variable. Features such as “headache”, “shortness of breath”, and “sore throat” exhibit high positive coefficients, indicating a strong association with the outcome. In contrast, “age 60 and above” and “gender” have lower coefficients, suggesting a lesser impact. We then compare these coefficients with the results obtained from our combination of interpretation methods. “Sore Throat” emerges as the most frequently identified significant feature, consistently appearing across different interpretation techniques.

Our logistic regression model particularly underscores the importance of “Sore Throat” as a critical predictor, validating the broader analysis and comparison with the combination of all interpretation methodologies. The high coefficient for “Sore Throat” in the logistic regression model confirms its significance, as indicated by the other methods. The consistent appearance of “Sore Throat” in the top four features across various methods further supports its importance. This convergence of results strengthens our confidence in the validity of our methods and demonstrates that our predictive model accurately reflects the true relationships within the data. This validation contributes to our confidence in the significance of “Sore Throat” in our predictive analysis task, both for COVID-19 test outcomes and as an indicator of an immune response to the virus. A deeper analysis reveals that recent studies have identified sore throat not only as a predictor of COVID-19 test outcomes but also as a crucial indicator of an active immune response to COVID-19 [38,39].

These studies have found that individuals presented with sore throats were likely to lack a detectable antibody response to the SARS-CoV-2 infection. This symptom indicates that the patient’s body may be unable to effectively deal with COVID-19 and is more likely to experience a more severe case of COVID-19 as their immune system is unable to generate antibodies, potentially leaving them more vulnerable to the virus’s effects. This showcases the importance of recognizing Sore Throat as not only a predictor for COVID-19 test outcomes but also as a critical indicator of an inadequate immune response, which can have significant implications for disease severity and patient prognosis. This dual role further emphasizes the pivotal nature of Sore Throat in our predictive models and presents its relevance in understanding the disease dynamics.

As shown in (Figure S1, Table 1 and Table 2) 99.4% of the values of sore throat feature are 0′s, indicating a low occurrence among individuals in our data. Despite this, our method has proven its capability to detect abnormalities and unusual cases as important features of the predictor, as can be seen in the methods in (Figure 10 and Figure 11). This ability to detect sore throat as an important feature even when it occurred infrequently (less than 1%) highlights the robustness of our approach and its ability to create complex relations between features and predictions.

With our trust in the model’s predictive power solidified (Figure 9), we can confidently assert that sore throat emerges as a significant feature for the predictor, even though it stands as an unusual case in our dataset. Additionally, recent studies indicate that sore throat could be an indicator of a compromised immune system or insufficient antibody response [45,46]; we find further support for its importance. Notably, our findings from the second dataset that describes vaccination doses and vaccination side effects [47] show that approximately 5% of severe cases of sore throat led to hospitalization. as can be seen in Figure 14.

To better understand the ~5% of cases involving sore throat during hospitalization, we examined active symptoms and observed that 4 out of 5 individuals with a sore throat also had shortness of breath. The combination of sore throat and shortness of breath in these four cases that led to hospitalization suggests a severe respiratory tract issue that obstructs airflow, hindering and restricting breathing. This suggests that sore throat cases were part of more significant respiratory problems due to compromised immune systems, which is common in COVID-19 infections (Figure S6) [48]. This indicates a compromised immune system [49], as researchers have found that sore throat is the most common ear, nose, and throat symptom in COVID-19 [50]. When the immune system is weakened or overwhelmed by a viral infection like COVID-19 or as a side effect of the COVID-19 vaccine [40], it becomes less effective in fighting off pathogens and preventing the progression of respiratory symptoms. A sore throat typically occurs due to inflammation and irritation in the throat [51], which can be caused by viral or bacterial infections. Shortness of breath, on the other hand, is a sign of respiratory distress that often results from mucus or fluid buildup in the lungs or airway constriction [52]. The combination and complication of these two symptoms suggest that the immune system is struggling to control the COVID-19 infection, leading to the development of more severe respiratory issues. As the immune system’s defenses are compromised, the body becomes more susceptible to complications and potentially respiratory failure.

Severe cases may also arise from vaccine-related immune responses, such as Adult-Onset Still’s Disease (AOSD) [53,54] or cytokine storms [55,56], which can cause systemic inflammation and tissue damage. Our data suggest that 40% of hospitalized post-vaccination cases with a sore throat, accompanied by significant muscle and joint pain, can be indicative of AOSD. In some post-vaccination cases, severe symptoms like sore throat and joint pain may indicate AOSD, linked to an excessive inflammatory response triggered by the vaccine. Additionally, It is speculated that the vaccine, particularly in susceptible individuals, may induce excessive production of inflammatory cytokines storms that can lead to conditions like Polyserositis, causing pleural inflammation and fluid buildup, resulting in chest pain and difficulty breathing [57,58]. This highlights the need for personalized post-vaccination monitoring and potential additional booster doses to enhance vaccine effectiveness. Recent trends show fewer sore throat cases with increased vaccine doses (Figure 13) [59], aligning with studies linking vaccine side effects to efficacy [6,7,60,61,62]. This underscores the need to dive deeper into the occurrences of sore throat post-COVID-19 pandemic vaccination, recognizing that further data in this area could greatly benefit and enhance our knowledge. It is essential to emphasize the importance of more comprehensive data to supplement our existing framework despite the inherent biases in self-reported symptoms. As we transition beyond the COVID-19 pandemic, understanding vaccine efficacy and immune response to the virus becomes increasingly crucial. The continual collection and dissemination of reliable data among public entities and researchers remain vital in this context. Alongside advancing our comprehension of symptomatology in diagnosing the disease, the potential integration of new symptoms into forthcoming models should also be considered.

With this amount of complex data being collected during and after the pandemic, machine-learning models have become invaluable tools for analyzing patient information and identifying potential risk factors. However, the black-box nature of many machine learning models can hinder their adoption in clinical settings, where interpretability and transparency are crucial for building trust and making informed decisions.

Our work focused on developing an interpretable machine-learning approach to aid in the early detection and risk assessment of severe COVID-19 cases. Our method showcased its ability to help users and clinicians gain better insights into the data, directing them toward more complex issues that cannot be identified merely by visual inspection of the data. Although sore throat appeared to be an unusual case in our data, global interpretation methodologies were able to detect it as a highly influential feature (Figure 9). This indicates that if a sore throat is accompanied by any other symptom, it could signify a more severe case. Researchers have found that the presence of a sore throat symptom accompanied by either shortness of breath or muscle and joint pain indicates a severe case where the immune system might be compromised [2,45,46,53,54,55,63]. This finding is significant because it highlights potentially severe symptoms, enabling more accurate and early detection of critical cases of the virus and improving the management and containment strategies for COVID-19. Understanding these critical features ensures that healthcare resources are directed efficiently and patient care is optimized.

This shows why more research is necessary to explore these interpretation methods. Such research can highlight the difference between these methods, and by incorporating their combination, we gain insights into how features influence predictions and what features are important for prediction and classification, regardless of the method used. By doing so, clinicians can start to investigate why specific features were marked as important in the hope of revealing deep and complex relationships within the data. This represents why machine learning interpretability is crucial in the medical field, where any additional insight can aid clinicians in making more informed decisions and providing better care to patients.

Our method was able to reflect this by providing clear and actionable insights from complex datasets, which are otherwise difficult to interpret. By making the decision-making process of the machine learning model transparent, we ensured that the critical features leading to severe cases were easily identifiable. This transparency not only builds trust with users and clinicians but also enhances the overall effectiveness of the model in real-world applications. Ultimately, the interpretability of our method facilitates better decision-making, early intervention, and optimized healthcare resource allocation.

### Study Limitation

A key limitation of our study is the simplicity of the data, which lacked detailed indications of the severity or timing of each symptom. Additionally, the dataset was relatively small, which may limit the generalizability of our findings. Furthermore, the data were self-reported, which introduces potential biases and inaccuracies in symptom reporting. While we explored cases with multiple symptoms, such as Adult-Onset Still’s Disease, where sore throat is accompanied by significant muscle and joint pain, and reported hospitalization cases where shortness of breath and sore throat were reported, our analysis primarily focused on individual symptom contributions due to the model’s design and the available data. This may overlook potential interactions between symptoms that could be significant predictors. Despite these limitations, we were able to demonstrate that the method we developed can still be beneficial, offering valuable insights into feature importance and helping to identify key indicators within the data. This suggests that our approach has the potential to be applied effectively even with imperfect datasets, though further validation with more comprehensive and larger datasets is warranted. 

## 5. Conclusions

In conclusion, despite the limitations inherent in our datasets and model, we have developed a unique method for robust outlier case detection, identifying important and highly influential features in medical data using state-of-the-art ML feature importance methods. This method proved effective in predicting and indicating important symptoms like sore throat from the onset of COVID-19 symptoms. Our findings suggest that investigating these symptoms early on can lead to a deeper understanding of the complex aspects of the immune system’s response and vaccine efficacy. When a sore throat is accompanied by other symptoms like shortness of breath, it could indicate a more severe case where the immune system is affected and not a regular case of COVID-19. This is especially important when these combinations of symptoms occur after receiving vaccination, raising the question of the vaccine efficacy and side effects.

Our research addressed the challenge of ML model Interpretations and clear explanations in medical data prediction. We accomplished this not only by comparing different methods from two distinct domains of global and local interpretation but also by combining these methodologies into a clear and transparent approach, helping clinicians gain a better and easier understanding of the model predictive process. Our approach makes it easier for clinicians because they do not have to choose a specific interpretation method from the wide array available. Instead, they can look at the final generalized results, which highlight the most important features, regardless of the methods used. It also assists them in comparing different feature importance rankings. If a feature is highly influential, it will always be the most important feature in the majority of methods. However, if a feature changes rankings a lot across methods, it could indicate that this feature is not a trusted and reliable indicator for the prediction task.

Using only eight basic questions, our model was able to achieve this, demonstrating its potential for use alongside other considerations like performance or applicability and other diseases. This approach could greatly aid researchers and physicians in easily identifying and understanding complex relationships within values with abnormalities. Even with biased or limited data, our method shows promise in advancing medical and symptom-related research, offering valuable insights and easy interpretation of complex issues for further advice and patient care.

Looking ahead, the future holds immense promise for enhancing our method. Advancements in AI and ML methods offer opportunities for further refinement and sophistication. By integrating our outlier case detection with pre-trained models like Personalized Medic Net, we can gain a deeper understanding of complex medical relationships. Additionally, advancements in Explainable AI (XAI) will enable clearer justifications for flagged unusual cases, enhancing transparency and trust in the model’s decisions.

This evolution toward a more comprehensive system for medical anomaly detection holds great potential. It not only assists in early symptom detection, as demonstrated with COVID-19, but also contributes to understanding the underlying mechanisms of diseases. Ultimately, this can lead to improved patient outcomes, more effective treatments, and further advancements in medical and symptom-related research.

## Figures and Tables

**Table 1:** Contingency table of sore throat and COVID-19 test results.

Sore Throat	Test Results 0	Test Results 1	Total
0	82,014	6143	88,157
1	84	878	962
Total	82,098	7021	89,119

**Table 2:** Percentage of binary values per each parameter.

Parameters	% of 0′s	% of 1′s
Cough	85%	15%
Fever	92%	8%
Sore Throat	99%	1%
Shortness of breath	99%	1%
Headache	99%	1%
Age 60 and above	36%	8%
Gender	45%	47%
Contact With Confirmed	96%	4%
Corona Result	95%	5%

**Table 3:** Comparison of feature importance methods: strengths and weaknesses.

Method	Model-Agnostic	Local Explanation	Global Explanation	Importance Metric	Strength	Weaknesses
ShAP	Yes	Yes	Yes	Average marginal contribution	1. Consistent 2. Unified measure	1. Computationally intensive 2. Bias to correlative features
LIME	Yes	Yes	No	Locality-aware loss.	1. Model-agnostic 2. Individual predictions	1. Stability issues 2. Parameter selection
LGBM	No	No	Yes	Entropy gain	1. Efficient 2. Direct importance metrics	Specific to the LightGBM model
DiCE	Yes	Yes	Yes	Counterfactual explanations by loss	1. Actionable insights 2. Diverse counterfactuals	1. Computationally expensive 2. Bias to correlative features
Sensitivity permutation	Yes	No	Yes	Feature contribution	1. General method. 2. Feature impact analysis	1. May oversimplify interactions 2. Bias to correlative features
Gradients	No	Yes	No	Gradient importance	1. Applicable to neural networks 2. Uncovers complex patterns	1. Can be noisy 2. Model-sensitive
Activation Maximization (CAMs)	No	Yes	No	Feature contribution to node importance	1. Efficient 2. Focusses only on important neurons in the network	1. Limited to specific NNs 2. requires modification
Pruning	No	No	Yes	Feature-node Impact on model efficiency	1. Simplifies model 2. Improves speed	1. Requires experimentation 2. Risk of loss of important nodes

**Table 4:** Performance of ML model.

Performances	LGBM Model	Baseline
ROC AUC	0.90	0.50
PRC AUC	0.68	0.43
Accuracy	0.86	0.62
Sensitivity (True Positive Rate)	0.83	0.56
F1 Score	0.48	0.43
Specificity (True Negative Rate)	0.86	0.73

**Table 5:** Performance of the DL model.

Performances	Neural Network Model	Baseline
ROC AUC	0.89	0.53
PRC AUC	0.64	0.32
Accuracy	0.91	0.53
Sensitivity (True Positive Rate)	0.77	0.43
F1 Score	0.58	0.33
Specificity (True Negative Rate)	0.92	0.63

**Table 6:** AUC score for the LGBM classifier using the top four features of each interpretation method.

Feature Importance Method	AUC Score
ShAP	0.83
DICE	0.65
LIME	0.65
LGBM	0.67

**Table 7:** AUC score for the DL classifier using the top four features from each interpretation method.

Feature Importance Method	AUC Score
Sensitivity	0.85
Activation	0.74
Prune	0.61
Gradient	0.58

## Data Availability

Data is available online [8,11]. The Code in this research is available at the following link: https://github.com/ShadiKhoury/Integrating-Interpretability-in-Machine-Learning-and-Deep-Neural-Networks (accessed on 8 September 2024). The component developed was meticulously crafted with flexibility in its very essence. It stands as a testament to adaptability, designed to be dynamic in nature. Users are not confined to singular paths; instead, they hold the reins to choose from a multitude of options. With the incorporation of inbuilt classifiers, users can swiftly navigate through choices. Furthermore, the component offers the freedom to import existing classifiers or forge entirely new ones. Its inherent prompts serve as a foundation for creativity, empowering users to tailor their experience to their precise needs.

## Supplementary Materials

The following supporting information can be downloaded at https://www.mdpi.com/article/10.3390/v16121864/s1, Figure S1: Distribution of Binary Values Across Each Feature; Figure S2: Neural Network Architecture of our model with the bottleneck layer; Figure S3: ROC Curve for LGBM Classifier Using Top 4 Features Identified by Each Interpretation Method; Figure S4: ROC Curve for DL Classifier Using Top 4 Features Identified by Each Interpretation Method; Figure S5: Percentage of symptoms reported per age group; Figure S6: Symptoms overview for cases of hospitalization where sore throat was present; Table S1: Performance metrics of LGBM model with CI’s; Table S2: Performance metrics of DL model with CI’s.
