# Supplementary material for: Integrating Interpretability in Machine Learning and Deep Neural Networks: A Novel Approach to Feature Importance and Outlier Detection in COVID-19 Symptomatology and Vaccine Efficacy
Source: Viruses. 2024 Nov 29;16(12):1864. doi: 10.3390/v16121864 (PMC11680429; doi:10.3390/v16121864)
Supplement: Supplementary file 1 [file viruses-16-01864-s001.zip › viruses-3225377-supplementary.pdf]

Supplementary material

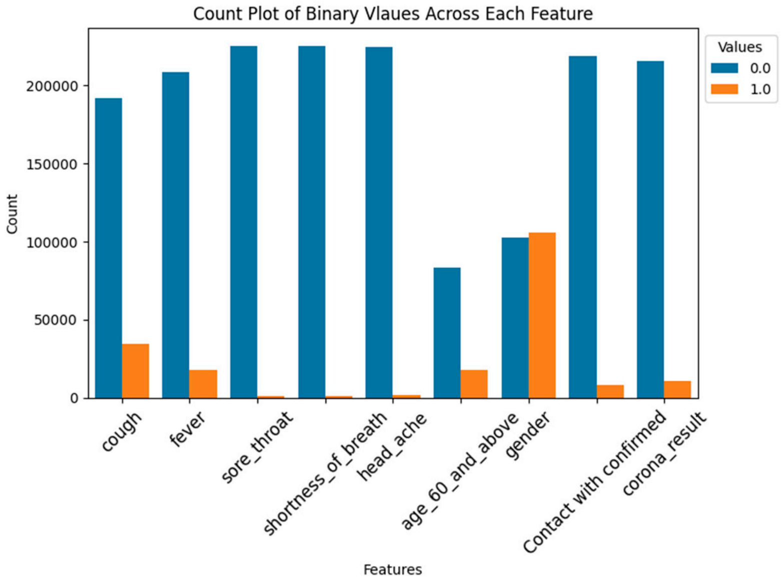

Figure S1: Distribution of Binary Values Across Each Feature. *The plot displays the distribution of binary values for each feature. Orange bars represent the count of instances where the feature value is 0 (not reported), while blue bars indicate the count where the feature value is 1 (reported).*

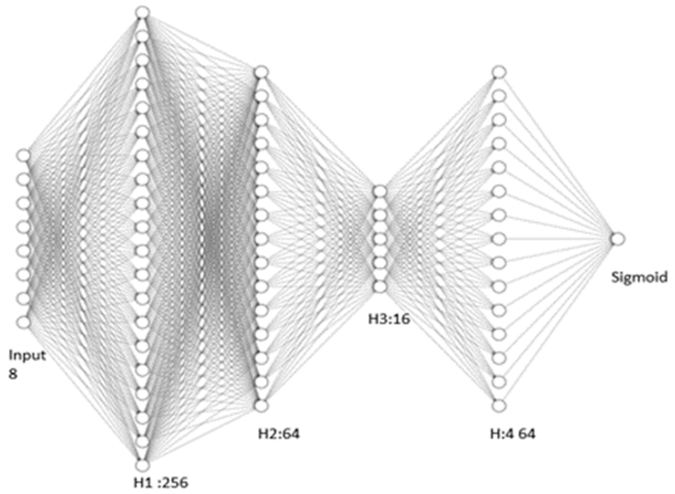

Figure S2: Neural Network Architecture of our model with the bottleneck layer. *The diagram illustrates the architecture of our neural network model, including the bottleneck layer. The plot shows the various layers of the network, with the bottleneck layer highlighted to emphasize its role in reducing dimensionality or complexity within the model.*

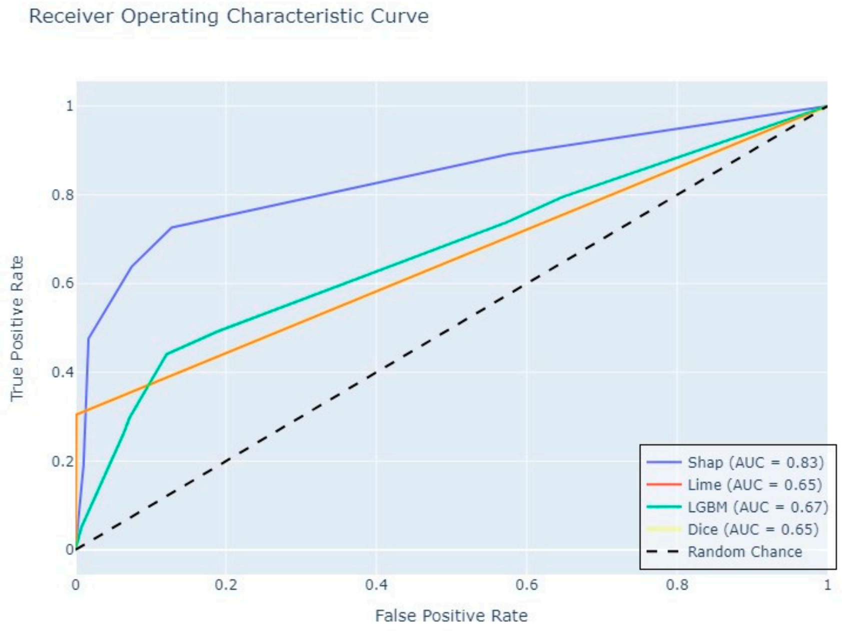

Figure S3: ROC Curve for LGBM Classifier Using Top 4 Features Identified by Each Interpretation Method

---

The Y-axis represents the True Positive Rate, and the X-axis corresponds to the False Positive Rate. Each plot in the graph represents a different method of feature importance, with each method yielding its respective AUC score.

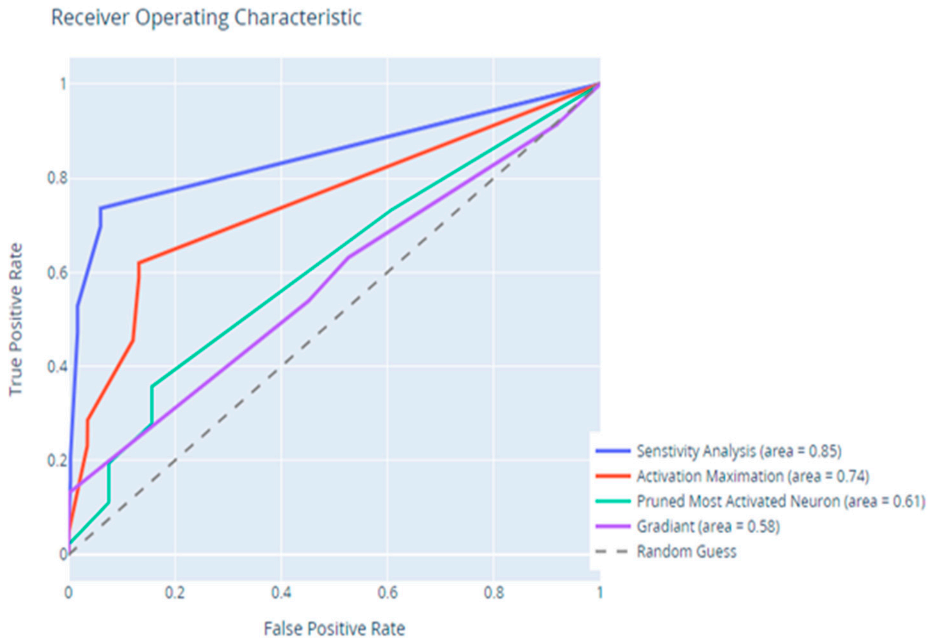

Figure S4: ROC Curve for DL Classifier Using Top 4 Features Identified by Each Interpretation Method

---

Where in this plot The Y-axis represents the True Positive Rate, and the X-axis corresponds to the False Positive Rate. Each plot in the graph represents a different method of feature importance, with each method yielding its respective AUC score.

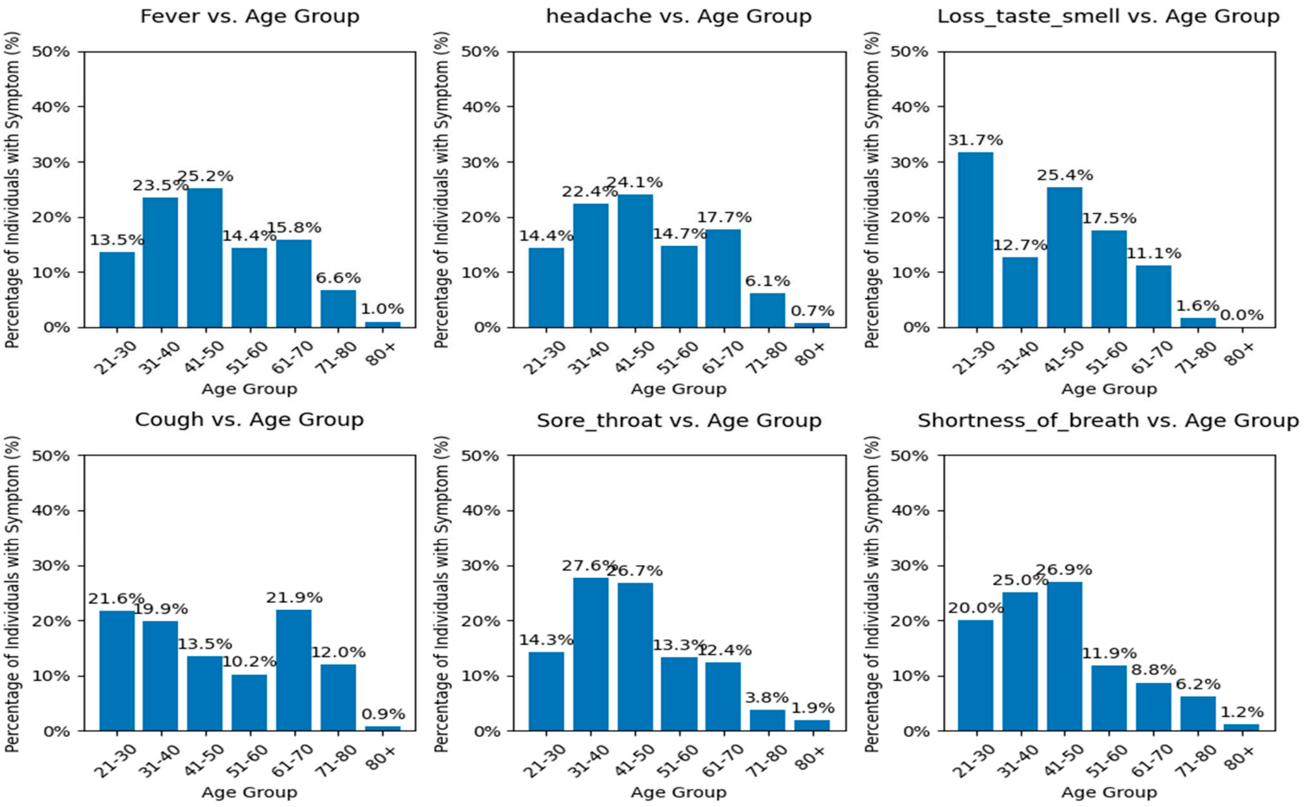

Figure S5: Percentage of symptoms reported per age group. *The plot displays the percentage of reported symptoms across different age groups. The x-axis represents the age groups, while the y-axis shows the percentage of individuals in each group who reported symptoms.*

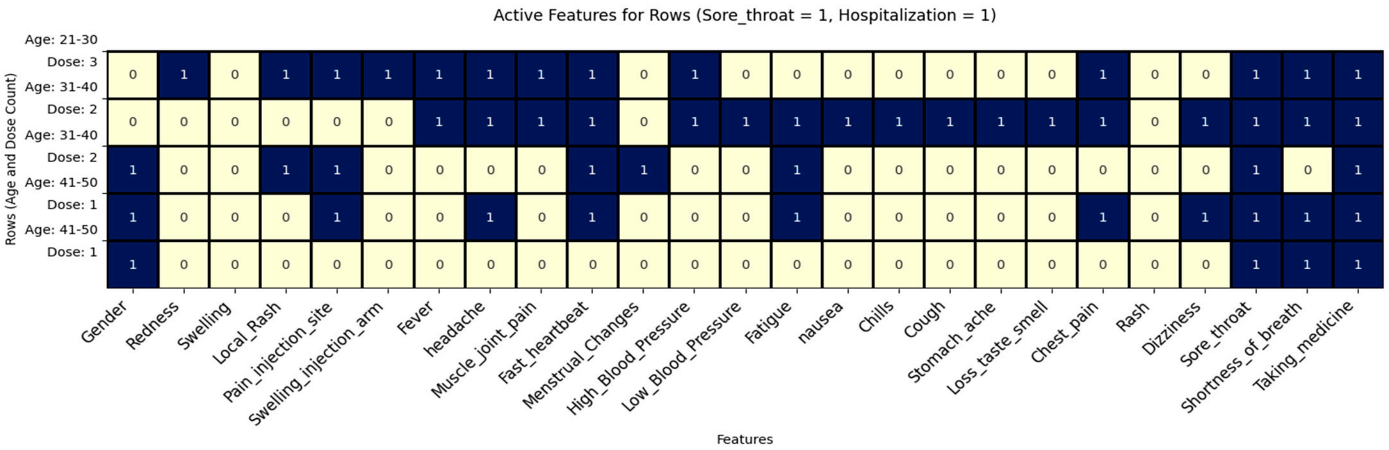

Figure S6: Symptoms overview for cases of hospitalization where sore throat was present. *The x-axis represents the various symptoms, while the y-axis includes the age group and number of doses administered for each hospitalization case where sore throat was present. Blue bars indicate the presence of the symptom (1), and absence of the symptom is denoted by 0.*

Performance metrics	*LGBM Model ± CI's*	*Baseline ± CI's*
**Accuracy**	**0.86 ± 0.003**	**0.62 ± 0.004**
**Sensitivity (True Positive Rate)**	**0.83 ± 0.01**	**0.56 ± 0.015**
**F1 Score**	**0.48 ± 0.008**	**0.43 ± 0.004**
**Specificity (True Negative Rate)**	**0.86 ± 0.003**	**0.73 ± 0.005**

Table S1: Performance metrics of LGBM model with CI's. This table includes accuracy, precision, recall, F1-score, and their respective 95% confidence intervals (CIs). The confidence intervals were computed using bootstrapping to ensure robustness and provide a reliable comparison with baseline performance**.**

Performance metrics	*LGBM Model ± CI's*	*Baseline ± CI's*
**Accuracy**	**0.91 ± 0.003**	**0.53 ± 0.004**
**Sensitivity (True Positive Rate)**	**0.77 ± 0.013**	**0.43 ± 0.016**
**F1 Score**	**0.58 ± 0.01**	**0.33 ± 0.005**
**Specificity (True Negative Rate)**	**0.92 ± 0.003**	**0.63 ± 0.006**

Table S2: Performance metrics of DL model with CI's. This table includes accuracy, precision, recall, F1-score, and their respective 95% confidence intervals (CIs). The confidence intervals were computed using bootstrapping to ensure robustness and provide a reliable comparison with baseline performance**.**
